# A Review on Lithium-Ion Battery Separators towards Enhanced Safety Performances and Modelling Approaches

**DOI:** 10.3390/molecules26020478

**Published:** 2021-01-18

**Authors:** Ao Li, Anthony Chun Yin Yuen, Wei Wang, Ivan Miguel De Cachinho Cordeiro, Cheng Wang, Timothy Bo Yuan Chen, Jin Zhang, Qing Nian Chan, Guan Heng Yeoh

**Affiliations:** 1 School of Mechanical and Manufacturing Engineering, University of New South Wales, Sydney, NSW 2052, Australia; ao.li@unsw.edu.au (A.L.); wei.wang15@unsw.edu.au (W.W.); i.decachinhocordeiro@unsw.edu.au (I.M.D.C.C.); c.wang@unsw.edu.au (C.W.); timothy.chen@unsw.edu.au (T.B.Y.C.); jin.zhang6@unsw.edu.au (J.Z.); qing.chan@unsw.edu.au (Q.N.C.); g.yeoh@unsw.edu.au (G.H.Y.); 2 Australian Nuclear Science and Technology Organization (ANSTO), Locked Bag 2001, Kirrawee DC, NSW 2232, Australia

**Keywords:** lithium-ion battery, separator, numerical modelling, battery safety

## Abstract

In recent years, the applications of lithium-ion batteries have emerged promptly owing to its widespread use in portable electronics and electric vehicles. Nevertheless, the safety of the battery systems has always been a global concern for the end-users. The separator is an indispensable part of lithium-ion batteries since it functions as a physical barrier for the electrode as well as an electrolyte reservoir for ionic transport. The properties of separators have direct influences on the performance of lithium-ion batteries, therefore the separators play an important role in the battery safety issue. With the rapid developments of applied materials, there have been extensive efforts to utilize these new materials as battery separators with enhanced electrical, fire, and explosion prevention performances. In this review, we aim to deliver an overview of recent advancements in numerical models on battery separators. Moreover, we summarize the physical properties of separators and benchmark selective key performance indicators. A broad picture of recent simulation studies on separators is given and a brief outlook for the future directions is also proposed.

## 1. Introduction

Pioneered by Yoshino in 1985 [1,2], lithium-ion (Li-ion) batteries have been commercialized and used ever since in the industry as an alternative source of energy. It is usually applied as an energy storage reservoir for renewable energies and commonly used in portable electronics and electric vehicles. Nonetheless, a Li-ion battery is less thermally stable in comparison with other battery systems. This has caused a significant amount of battery fires in recent years, which occurred in mobile phones, electric vehicles, and airplanes [3,4,5,6]. The Li-ion battery separator is one of the crucial factors affecting fire safety performance since it directly contributes to the thermal stability of the entire battery system.

**Figure 1:**
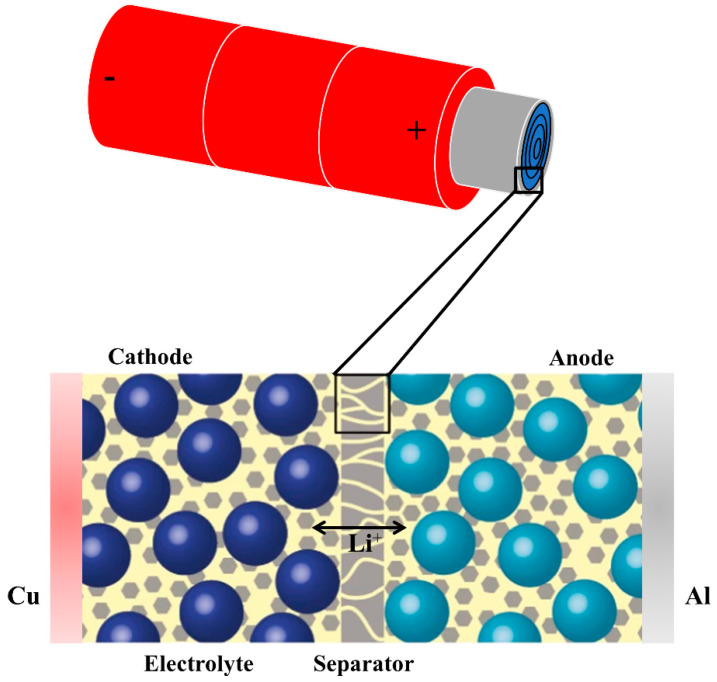
Schematic image of a separator in cylindrical Li-ion battery cell and a zoomed-in cross-section of the layered structure.

As one of the most important components in Li-ion batteries, the separator is placed between the anode and cathode [7]. The schematic diagram about a common separator applied in Li-ion batteries is shown in Figure 1, with the function of preventing physical contact between electrodes while serving as the electrolyte reservoir to enable ionic transport. There are no direct cell reactions in the separator, but the structure and properties of the separator play an essential role in determining the battery performance, including cycle life, safety, energy density, and power density, through influencing the cell kinetics [8]. A wide variety of factors should be considered while selecting appropriate separators for use in Li-ion batteries. Table 1 summarizes the general requirements that should be considered for Li-ion battery separators, and the detailed discussion has been provided by previous studies, such as development of membrane separators by Lee et al. [8], production process of separators by Deimede et al. [9], characterization and performance evaluation of separators by Lagadec et al. [10], and so on. These early reviews focused on the characterization methods for separator properties and manufacturing techniques for separators through experimental methods. Moreover, the application of the separator increases electrical resistance and takes up limited space inside the battery, which has a negative impact on battery performance. Therefore, reasonable utilization of a separator is of vital importance to improving the battery performance, which includes energy density, cycle life, power density, and fire safety.

In view of battery safety, the separator must be able to act as a blocking interface between the electrodes when an internal short circuit occurs, so that the thermal runaway is avoided [11]. Chemical and thermal stability, as well as shutdown function at the set temperature, should be the requirement for the separator. Considering the material price, current technology, and the trade-off relationship of the above properties, a comprehensive evaluation of the separator properties is required for separator selection. In order to improve the performance of separators and enhance the safety of Li-ion batteries, researchers have thus performed a lot of research work in recent years [12,13,14]. Furthermore, numerical modelling on the design and test of separators for improving battery abuse tolerance and performance is deemed a practical compromise in optimizing the separator in future battery systems. Compared with the experimental investigation on separators, numerical modelling is treated as an efficient and economic tool for the study of separators. Both the separator material properties and the various performances of the separator are able to be simulated and predicted by numerical models. In this paper, the current numerical studies of separators will be reviewed in terms of mathematical models, finite element analysis (FEA), and computational fluid dynamic (CFD) models, and molecular dynamic (MD) models. From the perspective of numerical study, we describe the separator performance based on its influence on the battery performance, including microstructure of separators, stress analysis for the separators, thermal and ion transport of separators, as well as degradation process of separators. Moreover, the relationship between separator properties and battery safety will be discussed based on the separator shutdown and separator breakdown. Based on this review, future research directions on the Li-ion battery separators will be discussed in detail.

## 2. Numerical Study of Separators

Separators must be chemically and electrochemically stable to the electrolyte and electrode materials in Li-ion batteries since the separator itself does not participate in any cell reactions. As a critical component inside Li-ion batteries under strongly oxidizing and reducing conditions when the battery is fully discharged and charged, separators should also be mechanically strong to withstand the high tension during the battery assembly operation. In terms of the properties and performances of the separator, related numerical studies of battery fire safety can be divided into separator shutdown and separator breakdown, which are reviewed in this section.

### 2.1. Numerical Methods

**Figure 2:**
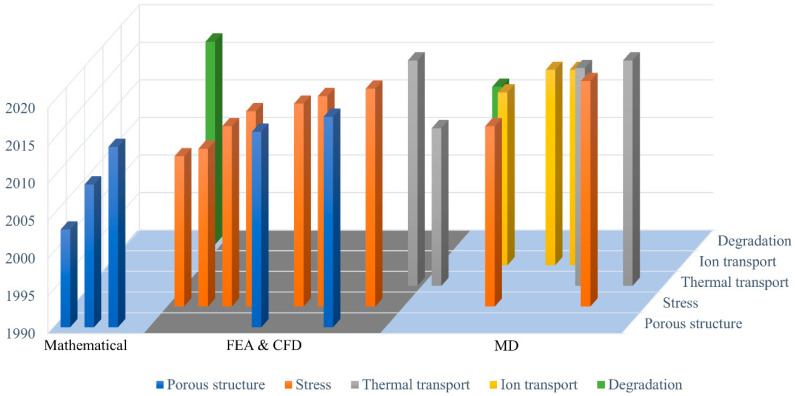
Summary of the reviewed papers for separators categorized by numerical methods and performances.

With the development of computer science, numerical simulations are gradually applied in many assessments of separator safety designs. Compared to standard experiments, numerical simulation validates experiment results with less physical resources and also reveals in-depth key performance parameters including temperature, pressure, electrochemical properties. Furthermore, we are able to visualize the battery system internally to effectively diagnose the problems that may lead to potential battery failures. The development of numerical battery models has facilitated better understanding of the underlying principles of the battery circuit and its associated influence towards the ambient environment. Figure 2 summarizes the reviewed numerical studies in this paper.

Mathematical models have been widely used in the battery property investigation and battery working procedure [15,16,17]. The development of a detailed mathematical model is important to design and optimize the batteries. Simulation results provide intuitive data on the performance of the battery. A suitable mathematical model can describe a few parameters which are not known experimentally and regulate parameter adjustment. For example, the direct experimental data for tortuosity or liquid-phase transport resistance is lacking, which can be simulated from mathematical models [18,19].

Finite Element Analysis (FEA) theories and methods originate from the need to solve complex elasticity and structural analysis problems in engineering [20]. This method has been developed and applied in studying the mechanical properties, and many FEA packages such as ABAQUS, LS-DYNA, RADIOSS were used to model the material structure. Computation fluid dynamics (CFD) is a practical tool to study different thermal fluid dynamic parameters and simulate multiple physics fields [21,22,23], and CFD makes it possible to use the equations governing a fluid motion for an extensive range of complex situations, providing both insight and quantitative predictions. CFD simulation can provide detailed information about the electrical and thermal field inside the battery that is often difficult to be assessed by experimental means. Model-based investigations promote theoretical understanding of battery physics beyond what is possible from experiments only.

Molecular dynamics (MD) simulations have been applied to understand the properties at the molecular-level and design chemical structures with high performance. There are variations of MD simulation models utilizing different chemical force-field based on their characterizing phenomena, for instance, pyrolysis [24], nucleation [25], material thermal/electrical properties [26], and so on. Moreover, MD simulations are used to predict the chemical interactions between different materials and understand numerous membrane properties. Table 2 summarized the numerical studies associated with separators, which are applied with numerical simulations, including mathematical models, FEA and CFD simulations, MD simulations, and so on.

### 2.2. Separator Shutdown

As shown in Figure 1, the location of the separator decides its primary function is to separate the anode and the cathode. The mechanical properties of separators are therefore very important for maintaining separation and Li-ion battery safety. Polyethylene (PE), polypropylene (PP), and PE/PP separators with pore sizes in the range of micrometres have been commercialized and widely used in Li-ion battery technology [49]. These microporous separators play a protective role during cell abuse. For example, if the temperature of the battery cell rises abnormally, separator shutdown occurs, which indicates that separators can provide a margin of safety to the device instead of leading to thermal runaway caused by the direct contact of electrodes. Numerical simulations can be carried out to study the microstructure and mechanical properties of the separator and to predict battery safety.

#### 2.2.1. Porous Structure

Microporous membranes are normally characterized by pore sizes in the micrometre scale and are mainly manufactured based on polyolefin materials, such as PE, PP, and their blends such as PE–PP, as they afford both excellent chemical stability and mechanical properties. High-density polyethylene (HDPE) and ultrahigh molecular polyethylene (UHMWPE) are also used for preparing microporous membranes [50]. Therefore, numerical study as a simplified analysis has been employed to evaluate the effect of separators in practice [16]. In mathematical modelling, the following empirical equation has been widely used.

(1) $$R_{s}=\varepsilon^{-\alpha }\cdot R_{0}$$

where *R_s_* is the resistance of the separator filled with liquid electrolyte, *R_0_* is the resistance of the native liquid electrolyte, *ε* is the void volume fraction in a separator, and *α* is the Bruggeman exponent. Separator morphology plays an important role in battery design and battery safety; therefore, numerical studies can provide better justification for the morphological parameters of separators for design and optimization.

Patel et al. [27] demonstrated models of porous networks to investigate the influence of particle shape and overall porosity on the liquid phase conductivity inside electrodes or separators used for Li-ion batteries. These models demonstrate that for batteries with high-rate performance, spherical or slightly prolate ellipsoidal particles should be preferred. Porous networks based on other particle morphologies however increase the tortuous path for ionic conductivity and result in either a significant increase of the exponent *α*, or a complete deviation from the power law. 

Thorat et al. [19] applied a mathematical model for an empirical relationship between porosity and the tortuosity of the porous structures. They concluded that the tortuosity-dependent mass transport resistance in porous separators and electrodes is significantly higher than that predicted by the often-used Bruggeman relationship. Moreover, Chen-Wiegart et al. [28] proposed a distance propagation method for calculating tortuosity with relatively low computation time from three-dimensional (3D) tomographic data.

Lagadec et al. [34] built an electrolyte-soaked separator model and studied the influences of the separator microstructure on the battery performance. The porosity *ε* and tortuosity *τ* of the polyethylene separators directly influence the transport properties (the concentration-dependent electrolyte *D_l_* and the concentration-dependent electrolyte *σ_l_*, calculated according to Nyman et al. [51]). The electrolyte conductivity decreased with the separator microstructure, and the potential drop can be thereby increased across the electrolyte-soaked separator. Based on their simulations, it is clearly illustrated that increasing the electrolyte conductivity and the transference number in separator membranes can improve the Li-ion battery performance, particularly at high current rates. Lagadec et al. [37] delivered an analysis of tomographic data of commercial separators. They demonstrated the extent to which Li-ion concentration gradients can be induced or smoothed by the separator structure. This is linked to the pore space connectivity, i.e., a parameter that can be determined by topological or network-based analysis of separators. 

#### 2.2.2. Stress Analysis

It is well recognized by the Li-ion battery community that stress plays an essential role in the performance of the separator. To enhance the battery separator’s performance, the stresses upon the separator in situ must be fully understood. Young’s modulus, which is a physical quantity parameter evaluating the anti-deformability of elastic materials subjected to external force, is applied to evaluate the mechanical performance of separators. In view of battery safety for Li-ion batteries, a larger elastic modulus enables the separator to sustain internal or external pressure and local stress. In order to evaluate the intercalation and thermal mismatch induced stresses in the separator, multi-scale multi-physics models have been proposed and developed [30,31,52]. Testing the mechanical properties of a separator in situ in a battery is one of the tasks in improving the performance of battery separators [53]. For an isotropic material, the mechanical stress has a constitutive relationship for the strain, which is given as [30]:

(2) $$\varepsilon_{ij}=\frac{1}{E}\left(\left(1+\nu \right)\sigma_{ij}-\nu \sigma_{kk}\delta_{ij}\right)$$

where *ε_ij_* is the strain component, *E* is Young’s modulus, *ν* is the Poisson’s ratio of the material, and *δ_ij_* is the Dirac delta function. Moreover, with the understanding of the mechanical properties of separators, battery safety performance can be estimated and optimized. Table 3 summarizes the numerical stress analysis results in this section.

Xiao et al. [30] developed a multi-physics, multi-scale model of a lithium-ion battery cell by using COMSOL. Their simulation results illustrate that the stress is affected by Young’s modulus of the separator, electrode particle size, separator wrapping patterns, and the pressure of the cell, and the local strain at the indented areas was much higher than the nominal strain of the separator.

**Figure 3:**
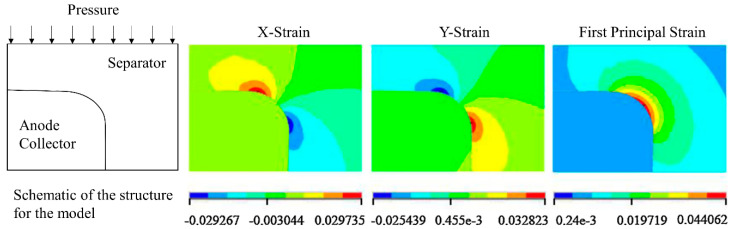
Schematic and contours of strain [m m^−1^] components in the separator near the corner [31].

Shi et al. [31] investigated the influences of some adjustable design parameters, including the effective friction, electrode particle radii, and thickness of the separator, on the stresses in the separator. It is concluded that the maximum Von Mises stress increased as increasing the thickness of the separator and the effective frictions between the separator and its adjacent electrodes. The stress analysis showed that the maximum stress in the separator always emerged at the area around the inner corner of the separator. In this case, the cell voltage at 4.2 V was assumed to be fully charged. The schematic of the structure represents the macro-scale 2D model with separator thickness of 25 µm and anode thickness of 45 µm. When the Li-ion battery was fully charged, the maximum stress was wrapped around the edge of the anode, as shown in Figure 3. In addition, with the same volume fractions of active materials, the particle radii had a negligible effect on the stress in the separator.

A multi-physics model was built by Wu et al. [32] to analyze the stress in the PP separator via COMSOL. The results showed that the effects of the intercalation and thermal expansion are coupled summations and hence must be considered concurrently. The type of the constitutive relationship of the separator affects the stress values. The calculated stresses in the separator with a viscoelastic material law were about a half of that estimated with an elastic law.

**Figure 4:**
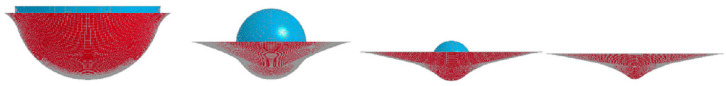
Punch test simulation with different punch sizes [33].

A finite element model of PE separator was developed in LSDYNA by Zhang et al. [33] based on the uniaxial tensile and through-thickness compression test data. The model succeeded in predicting the response of PE separator under punch tests with different sizes of punch head, including 1 inch (25.4 mm), 1/2 inch (12.6 mm), 1/4 inch (6.35 mm), and 1/8 inch (3.175 mm), which is shown in Figure 4. The model also correctly predicted the effect of anisotropic material on the shape and curvature of deformation in two planes of anisotropy. Furthermore, the anisotropic mechanical behaviour of the material can be analyzed by FEA models as well. Bulla et al. [54] developed a model to predict the anisotropic response of the PE separator due to deformation and failure by combining the novel failure criterion with Hill’s yield surface and a Swift–Voce hardening rule.

An image-based microstructure representative volume element (RVE) modelling method was applied by Xu et al. [35], which facilitates the understanding of the separators’ complex macro mechanical behaviour as a result of microstructural features. The proposed method successfully captures the anisotropic behaviour of the separator under tensile test and provides insights into microstructure deformation, such as the growth of voids. In this study, the imaging processing method and finite element simulation are successfully coupled to analyze the stress-strain relation of battery separators. Furthermore, Xu et al. [55] developed a microstructure modelling method to investigate the deformation patterns of the battery separator. Based on their results, the reason why the separator film turns transparent has two folds was explained. One is the material-level instability, and the other is the structure-level instability.

Lagadec et al. [36] characterized how the microstructural properties (including porosity, tortuosity, and permeability) of the separators change as a function of compressive strain and predicted the influence of these changes on the Li-ion transport through the separator by mechanical simulations. They also concluded that a given compressive strain negatively impacts the microstructure of PE separator more than that of a PP separator, because PE has a lower Young’s modulus, smaller pore sizes, and a more isotropic structure.

Xu and Bae [38] proposed a stochastic reconstruction algorithm to generate random but statistically equivalent 3D microstructure models for mechanical property analysis and uncertainty quantification. The proposed modelling method provides a tool to establish the “microstructure-property” relation, which can be considered as important separator design variables.

**Figure 5:**
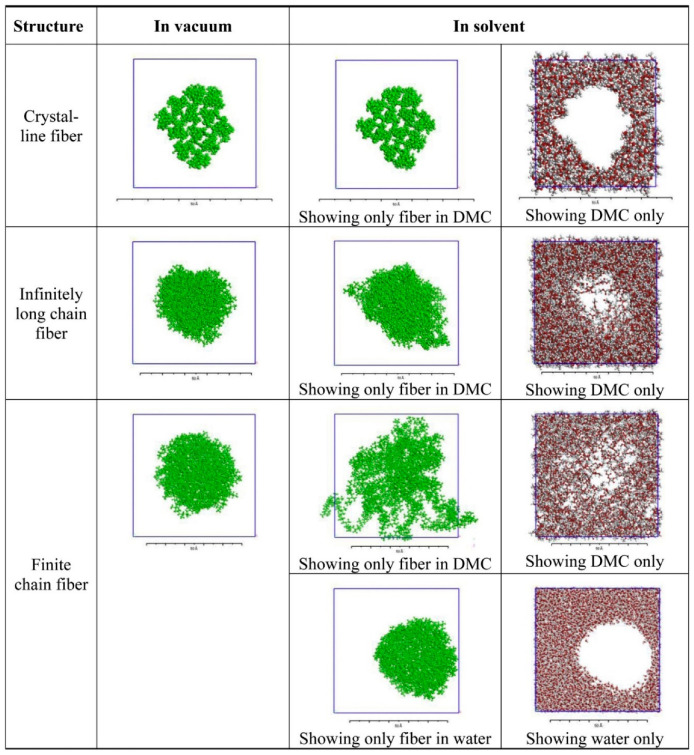
Relaxed structure for crystalline fibre, infinitely long chain fibre and finite chain fibre in vacuum, in DMC, and in water at zero strain [43].

In addition, from the molecular level, investigations of atomic interactions provide a deep understanding of the stress of separators in Li-ion batteries. Yan et al. [43] mapped the separator microstructure into discrete atomistic models of bulk crystalline phases and oriented amorphous nanofibers at different conditions such as in vacuum, water, and dimethyl carbonate (DMC) by using MD (See Figure 5). The mechanical responses of a porous PP separator in different media were found, which indicates that DMC can penetrate into the amorphous nanofiber and result in Young’s modulus reduction to one-tenth of its original value, while a polar solvent (e.g., water) can increase Young’s modulus by slightly squeezing the amorphous fibre due to the repulsive interaction.

**Figure 6:**
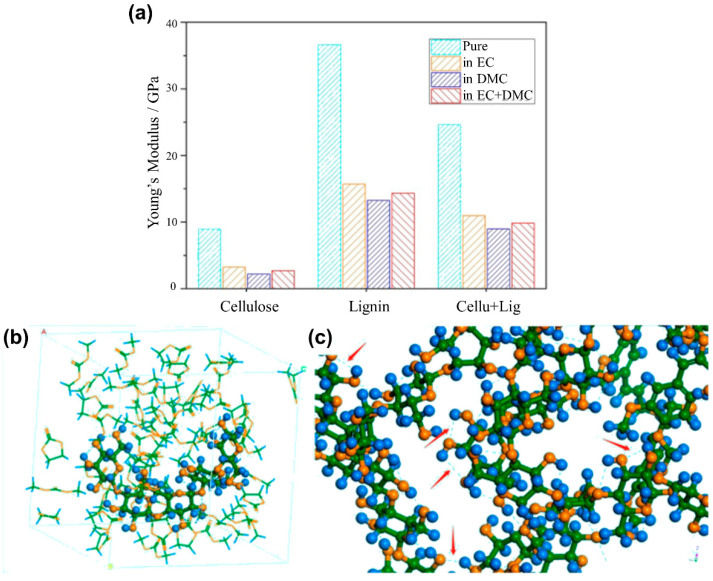
Simulation results from Xie et al. [47] (**a**) simulated Young’s modulus of molecular models under different environments, and distinct details in blended models: (**b**) deformed cellulose amorphous model in electrolyte solvents and (**c**) generated hydrogen bonds between cellulose and lignin molecules.

Xie et al. [47] successfully applied molecular simulation to unveil that the weakening of cellulose separator submerged in the electrolyte results from the deformed cellulose amorphous region and the promoting effect of adding lignin. The addition of lignin generates new hydrogen bonds between the cellulose and lignin molecules and subsequently form a larger fibrous network. The weakening phenomenon of cellulose separator immersed in the electrolyte is mainly caused by the deformation of the cellulose amorphous region, shown in Figure 6.

### 2.3. Separator Breakdown

During the battery working progress, it is possible that due to thermal inertia the temperature can continue to rise until the separator would melt and short the electrodes, leading to violent reactions and heat generation [56]. This phenomenon is called separator breakdown, which is one step of the Li-ion battery thermal runaway process. Therefore, the thermal properties of separators have a strong influence on battery safety. Numerical studies in this field can provide a better understanding of the separator breakdown mechanism and give reliable prediction results. Other related performances such as ion transport and the solid electrolyte interphase (SEI) degradation can be modelled as well by numerical methods.

#### 2.3.1. Thermal Transport

As mentioned in the Introduction, the separator acts as an essential role in improving battery safety performance. Low thermal transport in Li-ion cells and battery packs has been widely recognized as a critical technological concern that limits the use of Li-ion batteries [49,57,58]. Therefore, the focus for the current development of advanced Li-ion battery separators is to enhance the safety performance of the separator and/or facilitate the ionic flow through the separator during battery operation.

Standard MD simulation studies thermal transport and heat conduction across the molecular interfaces at the given length-scales (~a few nms). For example, MD simulations have been employed to model thermal transport across a variety of material pairs such as graphene-semiconductor heterostructures [59], Si/Ge interfaces [60], silicene/silica interfaces [61], graphene/phosphorene interfaces [62], etc. The thermal conductivity study on the electrodes and solid electrolytes have also been carried out, including equilibrium molecular dynamics [63] and nonequilibrium molecular dynamics simulation method [64].

Parviainen et al. [40] developed a new model, based on an existing MD code, to include resistive heating and electronic thermal conduction. The model accounts for dynamic changes of both tip geometry and temperature and gives an accurate and detailed view of the temperature development, including the temperature gradient in tips. For nanosized field emitters, it is critical to account for finite-size effects since both electric and thermal conductivity have a strong size dependence at this scale (height 13.1 nm and diameter 2 nm).

Non-equilibrium MD simulations were performed by Kawagoe et al. [46] on bulk amorphous polyacrylic acid with three polymer chain lengths to investigate the molecular mechanism of thermal energy transfer in heat conduction. The simulation results showed that the dominant mechanism of thermal energy transfer in polyacrylic acid (PAA) was intramolecular interaction. Consequently, the intramolecular interaction caused the thermal conductivity to increase as the polymer chain length elongated, which also increased the total thermal conductivity. The relation between thermal conductivity and the polymer chain length results in a saturation curve, which will lead to the characterization of thermal energy transfer in more complicated materials such as the layer-by-layer membranes.

**Figure 7:**
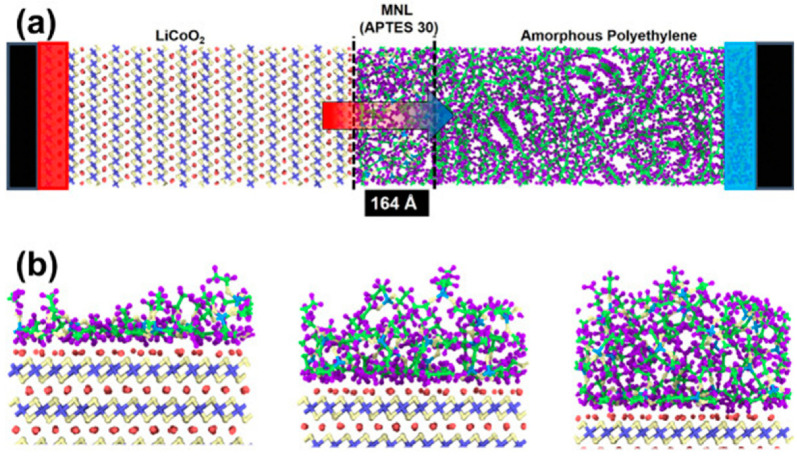
Representative images of the molecular assembly from Dhakane et al. [48]. (**a**) Molecular structure for simulation of a case with a bridging molecular layer (30 APTES molecules) at the LiCoO_2_-polyethylene interface. Hot reservoir (red rectangle on the left) and cold reservoir (blue rectangle on the right) maintained at 350 K and 250 K respectively are shown. (**b**) Schematics of the cathode-APTES interface for simulations with 10, 20, and 30 molecules.

Dhakane et al. [48] applied MD simulations for the calculation of thermal conductance across the cathode-separator interface with the interface force field in Figure 7. It is shown that molecular bridging at the interface results in up to 250% improvement in interfacial thermal conductance for the 3-Aminopropyl triethoxysilane (APTES) case. These results quantify the crucial role of the cathode-separator interface on thermal transport within the Li-ion cell, as well as the potential improvement in interfacial thermal transport by molecular bridging.

A two-dimensional electrochemical-thermal coupled model was developed by Li and Tan [39] for a 38120-type LiFePO_4_ Li-ion battery. Modeling results showed that the separator thickness strongly impacted battery energy density: the battery energy density dropped from 148.8 W h/kg to 110.6 W h/kg, while the separator thickness increased from 5 µm to 100 µm. The battery temperature rise and temperature difference dropped when both the separator thermal conductivity and heat capacity increased to 1 W m^−1^ K^−1^ and 3500 J kg^−1^ K^−1^, respectively.

#### 2.3.2. Ion Transport

During the working procedure of the Li-ion batteries, the transportation of ions plays an important role in battery performance. A separator membrane offers ion-conducting routes between electrodes and also electronically isolates the electrodes to prevent internal short-circuit failure, which eventually results in cell fire or explosion [65]. MD simulations have been developed in this area to explore the transport mechanisms and previous numerical studies investigated ion transport in the solid electrolyte [66], cathodes [67], and SEI [68,69]. The numerical simulations applied for separators will be reviewed in this section.

Vilčiauskas et al. [42] performed first-principles MD simulations to study proton conductivity at the fundamental molecular level, which represents a first step towards a more detailed understanding of the proton conduction mechanisms in realistic phosphoric acid-based polymer electrolyte materials. The solvation shell characteristics of the H-bond network are significantly over coordinated as compared to those in pure phosphoric acid. However, the lower density of H-bonds slightly increases the local molecular mobilities and artificially increases the proton diffusion coefficient.

Xu et al. [44] investigated ionic microporous zeolite membranes to overcome the challenge of the trade-off between ion selectivity and conductivity associated with conventional polymeric ion separators. They used the open-source software Packmol to construct the model and applied the LAMMPS package for running the simulations. The proton concentration in the zeolite structure (i.e., C_H+_) was determined to assist with understanding ion diffusion behaviour in the zeolite pores since the equilibrium values of C_H+_ are difficult to measure experimentally. This type of separator showed the ability to drastically reduce the self-discharge rates and enhance energy efficiencies.

Kim et al. [45] demonstrated the effect of the functional groups on the hydronium and hydroxide ions in hydrated poly(ether ether ketone) (PEEK) using MD simulations. The hydronium ion and hydroxide conductivity of the PEEK ion exchange membranes increased as the mole ratio of the functionalized moiety in PEEK increased. The diffusivity of the hydronium and hydroxide ions were calculated using their mean squared displacement in the simulation process via the COMPASS force field.

#### 2.3.3. Degradation

The most popular separator materials for Li-ion batteries with organic electrolytes are polyolefin materials [70]. However, the low melting point of polyolefins (135 °C for PE and 165 °C for PP) qualifies their utilization as a thermal fuse to shut down the cell by losing porosity and permeability if an over-temperature condition occurs. The main causes regarding separator degradation are typically traced to the lithium dendrite growth caused by separator pores, attack through the electrolyte, blockage of passageways in the separator over cycling, and structural degradation arising from elevated temperature or high cycle number [71].

A reduced-order capacity-loss model was applied by Jin et al. [29] to improve computational efficiency without sacrificing model fidelity. This model captures the two primary degradation mechanisms that occur in the graphite anode of a typical Li-ion cell: (a) capacity loss due to SEI layer growth, and (b) capacity loss due to isolation of active material. The model matches experimental capacity degradation results within a 20% error and 2400× faster than currently existing more complex physically-based electrochemical models.

**Figure 8:**
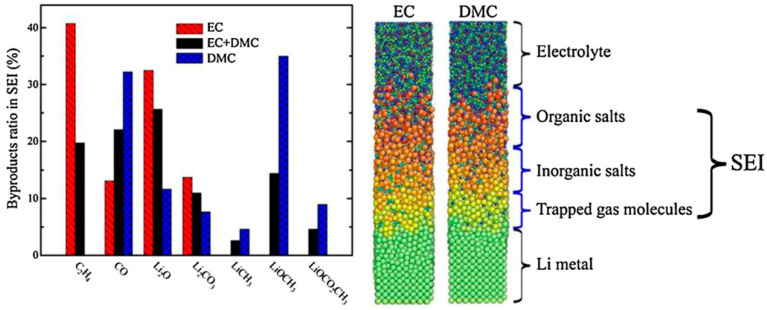
Distribution of the SEI components for different electrolytes (**left** chart). Atomic configurations from MD simulations; the components of the SEI are identified (**right**) [41].

Kim et al. [41] studied the formation and growth of SEI for the case of ethylene carbonate (EC), DMC, and mixtures of these electrolytes using molecular dynamics simulations. The simulation studied the distribution of organic and inorganic salts as a function of the distance from the anode surface in Figure 8, and the results show that inorganic salts are found closer to the anode surface while the region near the electrolyte–SEI interface is rich in organic salts.

## 3. Summary and Outlook

The separator is a crucial component in Li-ion batteries with the function of preventing physical contact between the positive and negative electrodes of the battery and stopping internal short while serving as the electrolyte reservoir to enable ionic transport. The ideal separator should not only have large electrolyte uptake for lowering the cell internal resistance but also have extremely thin thickness with strong mechanical strength, being electrochemically and structurally stable, as well as having a highly porous structure with great tortuosity to prevent the growth of dendritic lithium. In addition, the separator should be able to shut the battery down when overheating occurs for battery safety, as well as being cost-effective through the manufacturing process. However, it is challenging for practical separators to possess these ideal properties simultaneously, and therefore it becomes essential to balance different separator properties to achieve high-performance batteries. Moreover, the safety issue is still another obstacle for the separator and Li-ion battery applications.

In this paper, we have reviewed the recent numerical model advancements for Li-ion battery separators. It included mathematical and mechanical analytical-based simulation approaches such as FEA, CFD, and MD models. Through our summary, numerical simulation can be applied in the investigation of the properties of a separator and the prediction for the performance of the separators. Meanwhile, numerical studies not only provide a time-efficient and cost-effective way but also show a comprehensive understanding of the primary mechanism of the separator performance. Mathematical models describe certain parameters which are not known experimentally and provide the capacity of parameter adjustment. Mechanical models show a detailed microstructure of separators, and combining with FEA and CFD models, the properties of separators can be simulated and predicted. MD models demonstrate a more detailed view of the mechanisms, such as thermal propagation, ion transportation, and degradation. Furthermore, key mechanical design parameters such as Young’s modulus and average strain rate for various types of separator materials (i.e., polyolefin, PVDF, PP, PE, Cellulose/lignin, homogeneous solid medium) were analyzed through numerical simulation and characterization approaches. The development of robust, effective numerical tools to address the needs of fire safety of separators will be beneficial to the battery industry, providing a complementary design tool for safety engineering design and performance studies.

With the increasing demand of Li-ion batteries with high charge/discharge efficiency and energy density in the future, battery separators with high performances are required for both industrial and research purposes. Currently, the investigation on the separator materials and performances is mainly based on experiments. Numerical simulations can provide reliable results compared to experiments and contribute to study the mechanism of some effects, meanwhile, numerical simulations provide an efficient and economical way to develop the separator and battery system. To provide safer, more functional, and powerful separators, developing a novel Li-ion battery or battery system and optimizing the manufacturing process is critical. The following numerical investigations and development of models are recommended in the future: (i) an effective pre-system failure numerical tool that is able to diagnose the thermal propagation, short-circuiting, separator degradation; (ii) a novel thermal-runaway model for Li-ion battery systems that is able to incorporate multiple battery separator materials with different mechanical and physical properties; (iii) coupling of multi-scale simulation models to study the all-inclusive coupled internal/external phenomena of Li-ion battery fires; and (iv) the simulation findings can be inputted as useful parameters for machine learning algorithms.

## Figures and Tables

**Table 1:** General requirements for separators used in Li-ion batteries [8].

Parameter	Requirement
Chemical and electrochemical stabilities	Stable for a long period of time
Wettability	Wet out quickly and completely
Mechanical property	>1000 kg·cm^−1^ (98.06 MPa)
Thickness	20–25 µm
Pore size	<1 µm
Porosity	40–60%
Permeability (Gurley)	<0.025 s·µm^−1^
Dimensional stability	No curl up and lay flat
Thermal stability	<5% shrinkage after 60 min at 90 °C
Shutdown	Effectively shut down the battery at elevated temperatures

**Table 2:** The summary of the reviewed numerical studies for separators with model parameters.

Numerical Method	Model Parameters	Year	Ref
Mathematical model	Bruggeman exponent *α*	2003	[27]
	Tortuosity	2009	[19]
	Distance map, spatial distribution map, and histogram	2014	[28]
	Capacity loss, temperatures, and SOC	2017	[29]
FEA and CFD	Packing pattern, thickness variation, stress, and viscoelastic relaxation	2010	[30]
	Stress distribution, thermal effect, friction, particle radius, separator thickness	2011	[31]
	Principal stresses and Von Mises stress	2014	[32]
	Stress-strain curves and force-displacement curves	2016	[33]
	Porosity *ε*, tortuosity *τ,* and effective transport coefficient *δ*	2016	[34]
	Stress-strain curves, deformed shapes, and pores diameter	2017	[35]
	Strain, stress, node angle, voltage drop, and C-rate	2018	[36]
	Porosity, TP tortuosity, separator thickness, and connectivity density	2018	[37]
	Stress-strain curves	2019	[38]
	Thickness, porosity, energy density, heat generation rate, temperature, thermal conductivity, and heat capacity	2020	[39]
MD	Tip temperature, current density, and tip aspect ratio	2011	[40]
	Li density, SEI thickness, component ratio	2011	[41]
	Free energy, radial distribution functions, and proton transfer coordinate	2013	[42]
	Young’s modulus and density	2014	[43]
	Proton concentration (i.e., C_H+_)	2016	[44]
	Proton conductivity and ion exchange capacity value	2016	[45]
	Temperature, density, heat flux, and thermal conductivity	2019	[46]
	Young’s modulus	2020	[47]
	Interfacial thermal conductance	2020	[48]

**Table 3:** Stress analysis summary for separators used in Li-ion batteries.

Materials	Young’s Modulus (GPa)	Poisson’s Ratio	Average Strain (%)	Ref.
Polyolefin Poly(vinylidene fluoride) (PVDF)	0.2 0.05	0.35	−0.14 −0.035	[30]
A homogeneous solid medium	0.5	0.35	−0.40	[31]
PP separator Celgard 2400	0.1	-	-	[32]
PE microstructure PP microstructure	1.2 1.5	0	−0.40	[36]
PP	In vacuum/In DMC Crystalline fiber: 43.4/46.5 Infinitely long chain fiber: 0.66/0.07 Finite chain fiber: 0.29/0.01	-	-	[43]
Cellulose/lignin	Dry/Wet Pure cellu: 3.38/2.50 Lignin 2.5%: 3.90/3.58 Lignin 5%: 4.10/3.25 Lignin 7.5%: 4.23/2.98 Lignin 10%: 4.78/2.88	-	-	[47]
